# The minimally important difference of the Gastrointestinal Quality of Life Index for symptomatic gallstone surgery

**DOI:** 10.1007/s00464-020-08205-z

**Published:** 2021-01-04

**Authors:** Jason M. Sutherland, Carmela Melina Albanese, Trafford Crump, Guiping Liu, Ahmer Karimuddin

**Affiliations:** 1 Centre for Health Services and Policy Research, School of Population and Public Health, University of British Columbia, 201-2206 East Mall, Vancouver, V6T 1Z3 Canada; 2 Department of Surgery, University of Calgary, Calgary, AB Canada; 3 Section of Colorectal Surgery, Department of Surgery, University of British Columbia, Vancouver, Canada

**Keywords:** Cholecystectomy, Gastrointestinal Quality of Life Index, Minimally important difference, Patient-reported outcome

## Abstract

**Introduction:**

The Gastrointestinal Quality of Life Index (GQLI) is used to measure domains of health and symptoms among people with gastrointestinal disorders. The objective of this study is to calculate the smallest change in the GQLI that is perceived by patients as meaningful among a sample of English-speaking adult patients undergoing elective laparoscopic cholecystectomy for treatment of symptomatic gallbladder disease.

**Materials and methods:**

The study is based on retrospective analyses of a sample of participants completing the GQLI and the EQ-5D(3L) preoperatively and six months postoperatively in Vancouver, Canada. Patients are excluded if they are less than 19 years of age, cannot communicate in English, or reside in a long-term care facility. The MID is calculated for the GQLI’s domains using distribution and anchor-based methods.

**Results:**

Among eligible patients, the participation rate was 51%. The estimated MID for the overall GQLI value ranged between 4.32 and 11.44. There were no statistically significant differences in the GQLI’s MID values between sexes or age subgroups. There were statistically significant differences in the GQLI’s MID values by baseline health status.

**Discussion:**

This study should provide some comfort that the MID values used in discussing change in health and symptoms with elective cholecystectomy patients are robust to sex. Although the sample size may have been inadequate for age-based analyses, the study found large differences in MID values between age subgroups. Statistically significant differences in MID values based on preoperative health supports reporting MID values separately by baseline value. Further research should explore whether age-based differences in MID values exist using larger samples.

To understand patients’ well-being, questionnaires referred to as patient-reported outcomes (PROs) are increasingly being used to measure the patient’s perspective of his or her health, quality of life, and/or functional status [1, 2]. When administered preoperatively and postoperatively, PROs can be used to measure patients’ perspectives of surgical interventions [3]. For example, the Gastrointestinal Quality of Life Index (GQLI) measures quality of life using several domains relevant to people with gastrointestinal disorders, such as gallstones [4]. Surgical removal of the gallbladder (cholecystectomy, usually laparoscopic), is considered the most effective treatment for symptomatic gallstones [5, 6], which can produce highly uncomfortable symptoms such as severe abdominal pain, nausea, bloating, and constipation [7, 8]. English, German, Swedish, and Taiwanese versions of the GQLI have been validated for assessing the effects of gallstone disease and treatment on health-related quality of life (HRQoL) [4, 9–11], and the instrument is widely used to compare patients’ gastrointestinal-related health status pre and post cholecystectomy [8, 12, 13].

Previous research has reported that age, sex, and preoperative health status are associated with GQLI values; older patients with gallstones have shown poorer GQLI scores and smaller postoperative improvements [13]. The relationship between sex and health status is less clear: women presenting with gallstones have reported worse health status than men and greater improvement after cholecystectomy in unadjusted analyses, but similar improvement when controlling for age, diagnosis, comorbidities, and pre-intervention HRQoL [13], suggesting that age and pre-intervention health status may explain sex-based differences in health gains owing to cholecystectomy. However, women have a greater tendency to develop acute pain postoperatively than males, including after cholecystectomy [14, 15].

To infer whether cholecystectomy results in meaningful improvement in HRQoL, patients’ change in PRO score can be compared to the minimal important difference (MID). The MID measures the smallest change in a PRO score that is perceived by patients as meaningful [16–18] and serves as an important reference point for evaluating the effectiveness of therapeutic interventions [19, 20]. For example, if the context-specific MID of an instrument is ten points, patients reporting a change in PRO score of five points post surgery relative to their baseline score may not have experienced a meaningful change in health due to the intervention. The MID is relevant because tests of statistical significance are based on a function of sample size. Thus, while statistical significance describes whether a change has occurred, the change may not be meaningful from the patient’s point of view. Moreover, the MID provides a ‘benchmark’ against which to compare the effectiveness of interventions and untangles the concepts of statistical significance from meaningful clinical change as reported by the patient.

The MID can vary by population and context [1, 21], and to our knowledge no MID values of the GQLI have been reported for age, sex, or preoperative health subgroups of gallstone patients undergoing cholecystectomy, or for the English-language GQLI. To date, one study has estimated that the MIDs of GQLI domains among cholecystectomy patients range from 6.42 to 7.64 on a transformed scale of 0–100 [22]. However, this study needs very careful interpretation for several reasons. First, the MID was only calculated for improvements – not worsening – of health status. Second, the results are based on retrospective self-reported health and therefore may be subject to recall basis. Finally, the study is based on the Chinese version of the GQLI among a Taiwanese sample of patients and, thus, there may be some cultural interpretations that do not generalize to other countries or populations.

To address this gap in the literature, the primary purpose of this study is to calculate the MID for the domains and total score of the GQLI among a sample of English-speaking patients undergoing cholecystectomy for gallbladder disease. This study hypothesizes that the MID varies between sex, age, and preoperative health subgroups. Such knowledge can inform clinicians’ interpretation of GQLI scores to better identify candidates for cholecystectomy based on baseline health, expected improvement from cholecystectomy, and the appropriate subgroup MID, and can establish MID values for research or clinical trial purposes [20].

## Material and methods

### Patient recruitment and PRO collection

This study of the MID values of the GQLI is based on retrospective analyses of an existing cohort of cholecystectomy patients for treatment of symptomatic gallstones in Vancouver, Canada. Prospectively, sequential patients scheduled for elective cholecystectomy of 12 general surgeons practicing in four hospitals were identified and contacted to participate. Patients’ exclusion criteria included being younger than 19 years of age, unable to communicate in English, or residing in a long-term care facility.

Patients were contacted preoperatively by phone or mail between October 2014 and February 2019 [23, 24]. Participants completed a survey package which included the GQLI [4, 8, 10, 25] and the EQ-5D(3L) [26, 27] preoperatively. Participants completed the same PROs postoperatively, six months following their surgery, and postoperative data collection occurred until October 2019. In order to reduce loss to follow-up, participants who did not return their postoperative PROs were contacted via phone or email, reminding them to return their survey package.

This secondary analysis of participants’ PROs and administrative data was approved by the Behavioral Research Ethics Board of the University of British Columbia.

### The GQLI

The GQLI has thirty-six items asking patients about their gastrointestinal symptoms and interference. There are five domains of measurement of the GQLI, with nineteen items asking about symptoms, five items about emotions, seven about physical functions, four about social functions, and one item that relates to medical treatment effects [22]. Each of the 36 items has five response subgroups ranging from “all of the time” to “never” with responses coded from 0 to 4, respectively [4]. Individual item scores are summed to produce a total score ranging from 0 (worst health status) to 144 (best health status) [4]. Domain scores are calculated by dividing the sum of the domain’s items’ responses by the number of items in the domain [13].

The GQLI has been validated for assessing the effects of gallstone disease and treatment on patients’ quality of life [4, 10]. Previous studies have reported improvement in the GQLI total score and all domain values following cholecystectomy among patients with symptomatic gallstones [8, 10, 25].

### The EQ-5D(3L)

The EQ-5D(3L) is a five-item instrument that measures general health status [26, 27]. The instrument measures five domains, including: mobility, self-care, usual activities, pain/discomfort, and anxiety/depression. Each item’s response ranks the severity of problems: no problem, some problems, and severe problems. Combinations of the items’ responses are associated with health state utility values, a preference-based measure of health that ranges from values less than zero (worse than death) to 1 (full health) [28, 29]. Canadian-based utility values are available, derived from a sample of Canadians and independent of this study [30].

### Analysis of patient demographics

Participants’ PROs were linked with hospital discharge summary data. From the hospital discharge, participants’ age, sex, and comorbidities were ascertained. Because of the associations between age and GQLI score, as well as score change [13], age was categorized into four groups ranging from “younger than 50 years” through “older than 70 years”, as summarized in Table 1. Baseline health was categorized into four subgroups based on GQLI total score quartiles, as shown in Table 1. Age and baseline health subgroups were selected to keep sample sizes approximately equal between groups. Using the comorbidities, the Charlson comorbidity index [31] was calculated as an integer-valued representation of participants’ morbidity burden.

**Table 1:** Demographic and clinical characteristics of participants

Characteristic	All (100%)	Sex		*p-*value
		Female	Male	
	*n* = 188	*n* = 138 (73%)	*n* = 50 (27%)	
Age, mean (SD)	58.73 (13.33)	57.70 (13.22)	61.56 (13.36)	0.08
Age, *n* (%)				
< 50	50 (26.6)	40 (29.0)	10 (20.0)	–
50–60	46 (24.5)	35 (25.4)	11 (22.0)	–
61–70	61 (32.4)	45 (32.6)	16 (32.0)	–
> 70	31 (16.5)	18 (13.0)	13 (26.0)	–
Baseline GQLI total score	*n* = 169	*n* = 126	*n* = 43	–
< 96	41 (24.3)	32 (25.4)	9 (20.9)	–
97–112	46 (27.2)	34 (27.0)	12 (27.9)	–
113–125	43 (25.4)	34 (27.0)	9 (20.9)	–
> 125	39 (23.1)	26 (20.6)	13 (30.2)	–
Charlson Comorbidity Index, mean (SD)	0.11 (0.39)	0.07 (0.30)	0.24 (0.56)	0.04
0	172	131	41	–
1 or greater	16	7	9	–

The demographic characteristics of the sample of participants was summarized using means and percentages. Participants’ average change in GQLI scores and standard deviation were calculated for the overall sample. Differences between baseline and postoperative scores were tested using t-tests; the distribution of the scores was assessed visually and found to be approximately normally distributed. The MIDs of the GQLI overall score and its five domains were calculated for the overall cohort as well as age and sex subgroups, using the described methods.

### Estimation of the MID

There are two common approaches to estimate the MID of a PRO instrument known as anchor-based methods and distribution-based methods [1, 18, 32]. Distribution-based methods are based solely on the observed distribution of patients’ PRO values and represent observed change in the form of a standardized metric but provide no direct information about the MID [1]. They are commonly based on the sample’s baseline standard deviation (SD) or effect size [1, 18, 33]. In contrast, anchor-based approaches compare changes in PRO values to an external ‘anchor’ that can identify patients whose health has changed to a small but meaningful degree and which has a nontrivial association (correlation ≥ 0.30) with the PRO of interest [1]. One commonly used anchor is the level of change in an alternative PRO measure that corresponds to the minimal change perceived as meaningful in the target population (*i.e.,* the alternative PRO’s MID) [1, 18].

Given the heterogeneity of methods, even within the same sample of patients, different MID estimates can be generated [1]. To select the ‘best’ measure, or to narrow the range of plausible MID values, researchers can synthesize estimates from different estimation methods, experience from clinical trials, and conceptual understanding of the relationship between the chosen anchor and the PRO measure to narrow the range of MID values; when doing so, it has been suggested that anchor-based methods be assigned the highest priority since they take into account patient perception of health even though distribution-based approaches can be a useful starting point to detect a meaningful difference [1]. Whenever possible, sensitivity analysis encompassing multiple approaches is recommended [1].

To determine a plausible range of MID values, this study used both distribution- and anchor-based approaches to calculate the MID for the GQLI overall score and its five domains [34]. One distribution-based approach we used is referred to as the effect size method. It relies entirely on the standard deviation of baseline data, and the MID is taken as either 0.2 or 0.5 times the mean standard deviation of the sample’s baseline scores [35], known, respectively, as the *small* effect size method and the *medium* effect size method. Based on some empirical and psychophysiological evidence, some researchers argue that half a standard deviation (*i.e.,* the medium effect size) is a universal estimate of the MID [1, 18], while others acknowledge that half a standard deviation is a conservative estimate that is likely to be clinically meaningful, but does not necessarily correspond to the *minimal* important difference [36]. The second distribution-based approach we used is the standard error of the mean (SEM) method. Using the SEM method, the MID is taken as the product of the sample’s GQLI score standard deviation at baseline and the square root of one minus the relevant GQLI domain’s reliability [18, 37]. Estimates of reliability were extracted from the literature and utilize whichever of Cronbach’s alpha or intraclass correlation was available [10].

We refer to the anchor-based approach we used as the *regression method* as it consists of employing linear regression to estimate a line of best fit, with the anchor (change in EQ-5D(3L) score) as the independent variable and change in PRO score for which we wish to estimate the MID (GQLI) as the dependent variable [38, 39]. The change in EQ-5D(3L) utility value was chosen as the anchor because its correlation with the GQLI score change in most strata is near, or exceeds, 0.30 (results not shown) [1] and has precedents in related research [40–42]. The change in GQLI score when the anchor was set at the MID value of the EQ-5D(3L) was taken as an estimate of the GQLI’s MID. Because MID estimates for the EQ-5D(3L) among gastrointestinal surgery patients have not been published, we selected a value near the mid-range of MID estimates measured in patients with various conditions undergoing various interventions and took the MID of the EQ-5D(3L) to be 0.10 [34, 43]. A sensitivity analysis was performed to test whether MID estimates would differ if the EQ-5D(3L) MID was 0.04, the average of the total smallest health transitions defined by the instrument’s multi-attribute health classification system [41].

Each method to estimate the MID was repeated for sex, age, and baseline health subgroups. For each sex and age subgroup, the standard deviation of the subgroup’s MID was calculated using a bootstrap sampling approach where repeated samples were drawn from the original data and the MID was recalculated from each; the standard deviation of the empirical distribution of MID estimates was used as the subgroup’s standard error. To compare MID values between sex subgroups, a two-sample *t*-test was used to calculate the test statistic and *p*-value comparing the MID estimates, using the standard error derived from bootstrapping. To test for MID differences between age subgroups, the one-way analysis of variance (ANOVA) and Tukey’s test with Bonferroni’s correction to account for multiple comparisons between age subgroups were used (corrected *α* = 0.008). The distribution of baseline and postoperative scores were assessed visually and found to be approximately normally distributed. To test for MID differences between baseline health subgroups, ANOVA was used. MID values and standard errors for each subgroup as well as test statistics and p-values for all comparisons are reported.

All analyses were performed using R statistical software, version 3.4.1 (R Foundation for Statistical Computing, Vienna, Austria) [44].

## Results

Among the 647 cholecystectomy surgery patients eligible to participate, the rate of participants completing the preoperative survey was 51%. Then, among the 330 participants that completed the preoperative PROs, 57% of the 330 also completed the PROs postoperatively. This resulted in 188 participants (among 647 eligible) that completed the PROs preoperatively and postoperatively. Participants were, on average, three years older than non-participants (*p* < 0.01; results not shown) though no other differences in observable characteristics between participants and non-participants were detected.

As shown in Table 1, the mean age was 58 years and the majority of participants were female (73%). Most females had zero comorbidities and the average comorbidity burden was higher among males (*p* = 0.04). The most common comorbidity was hypertension. While not statistically significant (*p* = 0.08), there was some evidence that males were, on average, older than females. There were no obvious differences in preoperative health between males and females, except that a greater proportion of males (30%) than females (20%) reported GQLI total scores greater than 125.

The results of Table 2 show that among the overall cohort, there was a statistically significant difference in the mean GQLI total score between preoperative and postoperative measurements, consistent with improvement (*p* < 0.001). Statistically significant improvements in the mean scores of each GQLI domain (*p* < 0.001) and in the mean EQ-5D(3L) utility value (*p* < 0.001) were also observed.

**Table 2:** Pre and postoperative GQLI mean and standard deviation (SD)

Patient-reported outcome	Preoperative		Postoperative		*t*-stat	*p*-value
	Mean	SE	Mean	SE		
GQLI instrument total	106.97	1.80	117.2	1.58	7.22	< 0.001
GQLI—domain						
Symptoms	58.62	0.84	63.39	0.75	6.45	< 0.001
Emotions	14.27	0.30	16.57	0.25	8.36	< 0.001
Physical function	18.58	0.46	20.22	0.43	4.61	< 0.001
Social	12.91	0.24	13.81	0.21	3.84	< 0.001
Medical treatment	3.24	0.08	3.66	0.06	4.97	< 0.001
EQ-5D health state utility value	0.85	0.01	0.91	0.01	6.04	< 0.001

The estimated MIDs for the GQLI total score and domains using four different approaches (small effect size, medium effect size, SEM, and regression method) are shown in Table 3. The estimated MID for the GQLI total score ranged from 4.34 (in males, using the small effect size method) to 11.78 (in females, using the regression method). There were no statistically significant differences in the MID of the GQLI total score between sex subgroups (*p* > 0.05).

**Table 3:** Comparisons of the MID between females and males for the GQLI overall and domain score

GQLI domain	Overall	Males	Females	*t*-stat	*p*-value
Participants (*N*)	*N* = 188	*N* = 50	*N* = 138		
MID Distribution-based effect size, small (0.2* SD)					
Symptoms	2.19 (0.13)	2.11 (0.20)	2.21 (0.16)	− 0.17	0.87
Emotions	0.79 (0.04)	0.80 (0.08)	0.79 (0.04)	0.03	0.98
Physical function	1.22 (0.06)	1.17 (0.12)	1.24 (0.07)	− 0.16	0.87
Social function	0.64 (0.03)	0.63 (0.07)	0.64 (0.03)	− 0.03	0.98
Medical treatment effects	0.21 (0.01)	0.19 (0.02)	0.22 (0.02)	− 0.15	0.88
Instrument total	4.43 (0.25)	4.34 (0.47)	4.47 (0.28)	− 0.15	0.88
MID distribution-based effect size, medium (0.5*SD)					
Symptoms	5.48 (0.34)	5.28 (0.51)	5.54 (0.41)	− 0.27	0.79
Emotions	1.97 (0.09)	2.01 (0.20)	1.96 (0.10)	0.09	0.93
Physical function	3.06 (0.15)	2.92 (0.29)	3.10 (0.17)	− 0.27	0.79
Social function	1.60 (0.07)	1.57 (0.17)	1.61 (0.08)	− 0.08	0.94
Medical treatment effects	0.52 (0.03)	0.47 (0.06)	0.54 (0.04)	− 0.22	0.83
Instrument total	11.09 (0.62)	10.85 (1.18)	11.16 (0.71)	− 0.23	0.82
MID distribution-based SEM 1 SEM = (SD * $\sqrt{1-\mathrm{r}}$)					
Symptoms	3.64 (0.22)	3.50 (0.34)	3.67 (0.27)	− 0.22	0.83
Emotions	1.63 (0.07)	1.66 (0.17)	1.62 (0.09)	0.08	0.94
Physical function	2.45 (0.12)	2.34 (0.23)	2.48 (0.14)	− 0.23	0.82
Social function	1.53 (0.07)	1.51 (0.16)	1.55 (0.08)	− 0.08	0.94
Medical treatment effects	0.65 (0.04)	0.58 (0.07)	0.67 (0.05)	− 0.26	0.80
Instrument total	6.27 (0.35)	6.14 (0.67)	6.32 (0.40)	− 0.17	0.87
MID anchor-based regression method					
Symptoms	5.10 (0.74)	4.21 (1.18)	5.39 (0.90)	− 0.82	0.41
Emotions	2.60 (0.29)	2.81 (0.51)	2.56 (0.33)	0.27	0.79
Physical function	2.12 (0.37)	1.85 (0.78)	2.21 (0.42)	− 0.33	0.74
Social function	1.05 (0.25)	1.00 (0.50)	1.07 (0.27)	− 0.08	0.94
Medical treatment effects	0.49 (0.09)	0.35 (0.17)	0.54 (0.12)	− 0.35	0.73
Instrument total	11.44 (1.47)	10.14 (2.46)	11.78(1.71)	− 0.80	0.43

The *SD* is the standard deviation at baseline estimated using bootstrap sampling, *r* is Cronbach’s alpha for test–retest reliability except medical treatment effect where *r* is the estimated intraclass correlation. For the regression approach, the minimal change in EQ-5D-3L utility value is assumed to be 0.10. The standard error of each estimate is reported in brackets

Table 4 shows the estimated MID values for the GQLI total score and each domain using each of the four methods, stratified by age subgroup. No statistically significant differences in the MID of the GQLI total score were detected between age subgroups using the distribution-based approaches, and estimates were similar across age subgroups. Although the pairwise comparisons found no statistically significant difference between age subgroups’ MID estimates using the regression approach, differences between the numeric values were large (e.g., an 8.5-point difference between the MID of youngest participants and those aged 50–60 years).

**Table 4:** Estimated MID (standard error) by age subgroup and results of testing for differences in the MID between age subgroups estimated MID for each GQLI domain and overall

GQLI domain	< 50 years	50–60 years	61–70 years	> 70 years	Largest	Differences in means
	A	B	C	D	*T*-statistic	
Participants (*N*)	50	46	61	31		
Distribution-based effect size, small (0.2* SD)						
Symptoms	2.15 (0.30)	2.51 (0.30)	2.06 (0.20)	2.08 (0.20)	1.24	ABCD
Emotions	0.84 (0.07)	0.77 (0.07)	0.75 (0.07)	0.79 (0.10)	0.90	ABCD
Physical function	1.26 (0.12)	1.37 (0.13)	1.15 (0.12)	1.08 (0.16)	1.40	ABCD
Social function	0.69 (0.07)	0.61 (0.05)	0.61 (0.06)	0.61 (0.06)	0.92	ABCD
Medical treatment effects	0.24 (0.02)	0.18 (0.03)	0.22 (0.03)	0.19 (0.02)	1.76	ABCD
Instrument total	4.53 (0.44)	4.67 (0.55)	4.34 (0.42)	4.15 (0.54)	0.67	ABCD
Distribution-based effect size, medium (0.5*SD)						
Symptoms	5.41 (0.74)	6.27 (0.73)	5.16 (0.50)	5.19 (0.50)	1.25	ABCD
Emotions	2.10 (0.16)	1.92 (0.16)	1.88 (0.18)	1.96 (0.26)	0.91	ABCD
Physical function	3.14 (0.27)	3.41 (0.32)	2.88 (0.30)	2.69 (0.41)	1.38	ABCD
Social function	1.73 (0.18)	1.52 (0.13)	1.52 (0.15)	1.52 (0.16)	0.94	ABCD
Medical treatment effects	0.59 (0.05)	0.44 (0.08)	0.55 (0.07)	0.46 (0.05)	1.83	ABCD
Instrument total	11.34 (1.11)	11.68 (1.38)	10.85 (1.04)	10.38 (1.36)	0.67	ABCD
Distribution-based SEM 1 SEM = (SD * $\sqrt{1-\mathrm{r}}$)						
Symptoms	3.59 (0.49)	4.16 (0.49)	3.42 (0.33)	3.44 (0.33)	1.25	ABCD
Emotions	1.73 (0.14)	1.58 (0.14)	1.55 (0.15)	1.62 (0.21)	0.87	ABCD
Physical function	2.52 (0.22)	2.73 (0.25)	2.30 (0.24)	2.15 (0.33)	1.40	ABCD
Social function	1.66 (0.17)	1.45 (0.13)	1.46 (0.14)	1.45 (0.15)	0.98	ABCD
Medical treatment effects	0.73 (0.06)	0.54 (0.10)	0.68 (0.08)	0.57 (0.07)	1.73	ABCD
Instrument total	6.41 (1.37)	6.61 (1.70)	6.14 (1.28)	5.87 (1.68)	0.30	ABCD
Anchor-based regression method						
Symptoms	7.65 (1.65)	3.03 (1.59)	4.78 (1.24)	4.55 (1.59)	2.01	ABCD
Emotions	3.22 (0.55)	2.35 (0.62)	2.91 (0.51)	1.70 (0.64)	1.80	ABCD
Physical function	2.26 (0.78)	1.69 (0.74)	2.73 (0.53)	2.16 (0.99)	1.14	ABCD
Social function	1.12 (0.46)	0.62 (0.43)	0.54 (0.48)	2.63 (0.62)	2.66	ABCD
Medical treatment effects	0.75 (0.19)	0.23 (0.17)	0.69 (0.21)	0.18 (0.16)	2.29	ABCD
Instrument total	15.73 (2.96)	7.11 (3.07)	11.62 (2.32)	12.27 (3.40)	2.02	ABCD

*r* is Cronbach’s alpha and the minimal change in EQ-5D-3L utility value is assumed to be 0.10. Pairwise comparisons were calculated for each pair of age subgroups, adjusted for multiple comparisons

Table 4 also shows that no statistically significant differences in MID estimates between age subgroups were found for the symptoms, physical function, social function, or medical treatment effects domains of the GQLI (*p* > 0.008). The largest pairwise difference in estimated MID values between age subgroups for the Emotions domain was found using the regression method.

As shown in Table 5, for the GQLI total score and all except one GQLI domain, MID estimates were largest among the subgroup of participants with the lowest GQLI scores at baseline (*i.e.*, worst preoperative health) and decreased with improving preoperative health. The sole exception was the MID of the Emotions domain estimated using the regression method – the subgroup of patients who scored 91–112 preoperatively had a smaller MID than those who scored 113–125. The ANOVA provided evidence of statistically significant differences in MID estimates based on baseline GQLI total score using both the medium effect size and linear regression methods (*p* < 0.05).

**Table 5:** Estimated MID (standard error) by preoperative GQLI total score and results of testing for differences in MID between baseline health subgroups for the GQLI domains and overall score

GQLI domain	< 96 A		91–112 B		113–125 C		125–144 D		Largest *T*-statistic	Differences in means
	MID (SE)	*n*	MID (SE)	*n*	MID (SE)	*n*	MID (SE)	*n*		
Medium effect size										
Symptoms	4.97 (0.59)	41	2.51 (0.27)	46	2.00 (0.17)	43	1.63 (0.28)	39	41.47	A B C D
Emotions	1.73 (0.18)	41	1.36 (0.11)	46	1.19 (0.17)	43	0.81 (0.11)	39	28.18	A B C D
Physical function	2.28 (0.19)	41	1.73 (0.19)	46	0.98 (0.11)	43	0.89 (0.08)	39	40.84	A B C D
Social function	1.32 (0.14)	41	1.02 (0.10)	46	0.99 (0.13)	43	0.31 (0.05)	39	40.62	A BC D
Medical treatment effects	0.60 (0.05)	41	0.51 (0.06)	46	0.31 (0.06)	43	0.15 (0.04)	39	37.54	A B C D
Instrument total	7.16 (0.71)	41	2.51 (0.19)	46	2.00 (0.13)	43	1.96 (0.14)	39	62.98	A B CD
Linear regression										
Symptoms	10.72 (1.81)	36	5.27 (1.49)	39	3.66 (0.90)	36	− 0.51 (1.14)	33	33.62	A B C D
Emotions	4.49 (0.67)	36	2.22 (0.58)	40	2.26 (0.35)	36	0.70 (0.56)	34	28.64	A BC D
Physical function	4.72 (0.91)	36	1.68 (0.70)	40	1.36 (0.44)	34	− 0.07 (0.53)	34	29.68	A BC D
Social function	2.79 (0.58)	34	0.98 (0.45)	40	0.29 (0.37)	35	− 0.28 (0.25)	35	29.71	A B C D
Medical treatment effects	0.98 (0.24)	34	0.62 (0.19)	40	0.27 (0.12)	35	− 0.12 (0.11)	35	26.34	A B C D
Instrument total	23.91 (3.35)	34	10.68 (2.48)	39	7.76 (1.38)	34	0.08 (1.77)	31	40.27	A B C D

Results are shown for MID estimates produced using the medium effect size (distribution-based) and linear regression (anchor-based) methods. Pairwise comparisons were performed using the Tukey–Kramer test

The sensitivity analysis of the anchor value found that using an anchor value of 0.04 produced MID estimates between 12 and 64% smaller than when the value of 0.10 was used. Results of the sensitivity analysis showed evidence of MID differences between age but not sex subgroups (Appendices 1 and 2 in Tables 6 and 7). Differences in MID values between baseline health subgroups were still identified (Appendix 3 in Table 8).

## Discussion

The objective of this study was to calculate the MID of the GQLI among a sample of English-speaking adult participants and to report whether the MID values differed between sex, age, and baseline health subgroups. MID values for the GQLI total score and among English speakers undergoing cholecystectomy have not been previously reported in the literature. Furthermore, methodological results regarding sex, age, and baseline health-based differences in MID values fill an important gap in understanding change among several domains of health measured by the GQLI since the MID can vary with patient population. This is important summative research since the MID provides a ‘benchmark’ for evaluating interventions and untangles statistical significance from patients’ perceptions of their change in self-reported health.

Depending on the approach taken to calculate the GQLI’s overall MID, this study found considerable variation in MID estimates. Despite the range in MID values obtained by using various estimation methods, we observed some patterns; first, the small effect size method consistently produced MID estimates smaller than those obtained using all other methods. Also, consistent with previous reports [1, 18], MID estimates obtained using the anchor-based method and medium effect size method were generally similar; this result was observed among the overall cohort and both sex subgroups for many domains as well as the GQLI total score. Estimates using these two approaches were not as similar when participants were stratified by baseline health status.

When transformed to the same scale, our estimates of the MID of most GQLI domains were consistent with Shi et al.’s [22] MID estimates, providing additional support for our findings, and shown in Appendix 4 in Table 9. One exception to this consistency of result was found in the Emotions domain—our MID estimate was nearly two times larger. It is unlikely that baseline health status was the reason for this discrepancy since our sample’s baseline health was similar to that of Shi et al.’s [22].

An important finding for clinicians and patients is that the results found no difference in MID values between sexes for the GQLI total score or the instrument’s domains. Not only were MID differences not statistically significant, but the MID estimates were similar between males and females, suggesting it is unlikely that any meaningful differences in GQLI MID values exist between males and females. This finding is relevant to clinicians as it confirms that the GQLI is robust to the sex of the respondent, and that clinicians should not need to adjust MIDs for patient’s sex.

The findings for age subgroups were more complex to interpret; statistically, there were no differences in the GQLI’s MID between age subgroups. However, the results showed that the MID of the instrument’s total score differed by over 8 points between age subgroups, suggesting possible but inconclusive evidence of differences in MID values between age subgroups. Although this result was based on subgroups of 31 to 61 participants, it is possible that either the sample size was still too small to detect statistically significant differences, the Bonferroni adjustment was too conservative, or that participants in certain age subgroups experienced a wider range of outcomes (higher variance) than participants of other ages attributable to other, unmeasured, effects such as symptom severity. The implications of uncertainty by age subgroup is meaningful to clinicians; since other research has reported larger improvement in younger individuals [13], the patient’s expected benefits attributable to the cholecystectomy should assumed to be different by age patient’s age in directionality with the results presented in this study, with specific values determined by additional research.

We found strong evidence that the MID values of the GQLI differed by baseline GQLI values, which was consistent with expectations since previous research has reported larger quality of life gains among patients with worse health and smaller gains in patients with better preoperative health [13]. In our study, participants with higher GQLI total scores (*i.e.*, better health) preoperatively perceived smaller changes in health as meaningful compared with participants who scored lower preoperatively (*i.e.*, worse health).

Unexpectedly, we found that MID values estimated using the anchor-based approach among the highest scoring participants were negative; reflecting that among participants with the highest (best) preoperative health, gains in health-related quality of life may not accrue. Also, we found that the variance in GQLI scores was highest among participants with the worst baseline health; since distribution-based approaches produce MID values that are a function of the sample’s variance, this finding had a profound influence on the distribution-based MID estimates and provides support to the claim that the anchor-based approach, rooted in patients’ perspectives of their health, should be preferred when available.

Another important finding is that the value of the anchor can influence the results. In our study, halving the EQ-5D(3L) threshold value had negligible effect on the statistical significance, though the MID estimates varied greatly—demonstrating the sensitivity of this approach. This finding underlines the importance of estimating the MID within specific patient subgroups and reinforces that further research is needed to provide a better understanding of the EQ-5D(3L)’s use among elective cholecystectomy patients.

There are limitations to this study, as the sample size was less than 200 participants and follow-up for postoperative completion of PROs was less than 60%. Despite the participation rate, the only observable characteristic participants and non-participants differed by was age, with participants three years older, on average, than non-participants. Nonetheless, there is a risk that differences in unobservable characteristics, such as disease severity, could have introduced bias into our results. Additionally, the complexities of the age subgroup results suggest further research in larger samples is warranted. Also, this study applied a conservative approach to comparing subgroups; however, the study did not adjust for the fact sex and age subgroups were measured simultaneously, potentially lowering the threshold p-value further. In spite of these limitations, we found substantive evidence that the MID value varies by preoperative health status.

The findings of this study are important to inform the clinician or patient’s expectations regarding the effect of cholecystectomy on health and to inform thresholds for cholecystectomy effectiveness research. Based on our findings, we conclude that the MID is robust to sex. While we are inconclusive on whether MID values vary by age, clinicians should note that MID values vary based on patients’ preoperative health. Since distribution-based approaches to estimating the MID are heavily influenced by the sample’s variance versus the patient’s perspective of health, we conclude emphasis should be placed on MID estimates obtained using anchor-based approaches. Consequently, the ‘best’ MID estimates for the GQLI total score are: 23 among patients scoring less than 96 preoperatively; 10 among patients scoring between 91 and 112; 7 among patients scoring between 113 and 125; and 0 among patients scoring between 125 and 144.

## Appendix 1

See Table 6

**Table 6:** MID values estimated using the linear regression (anchor-based) method for males and females

GQLI domain	Overall	Males		Females		*t*-stat	*p*-value
		MID (SE)	*n*	MID (SE)	*n*		
Symptoms	3.98 (0.78)	2.94 (1.14)	36	4.34 (0.95)	113	− 0.97	0.33
Emotions	1.94 (0.28)	1.63 (0.52)	36	2.08 (0.30)	119	− 0.50	0.62
Physical function	1.33 (0.37)	0.66 (0.70)	36	1.59 (0.44)	118	− 0.87	0.39
Social function	0.68 (0.24)	0.62 (0.43)	37	0.70 (0.29)	116	− 0.09	0.93
Medical treatment effects	0.37 (0.09)	0.30 (0.15)	38	0.39 (0.12)	120	− 0.17	0.87
Instrument total	8.55 (1.48)	5.86 (2.26)	32	9.36 (1.75)	106	− 1.75	0.08

The EQ-5D threshold (anchor) was set to 0.04. Differences between males and females were tested using a *t*-test with alpha set at 0.05

.

## Appendix 2

See Table 7

**Table 7:** MID values estimated using the linear regression anchor-based method for each age subgroup

GQLI domain	< 50 years		50–60 years B		61–70 years C		> 70 yearsD		Largest *T*-statistic	Differences in means
	MID (SE)	*n*	MID (SE)	*n*	MID (SE)	*n*	MID (SE)	*n*		
Symptoms	5.37 (2.04)	38	2.40 (1.76)	39	4.20 (1.19)	48	3.09 (1.56)	24	7.90	A C BD
Emotions	2.23 (0.61)	40	2.02 (0.65)	41	2.17 (0.42)	50	0.80 (0.52)	24	10.00	ABC D
Physical function	1.33 (0.84)	40	1.19 (0.87)	41	1.57 (0.52)	51	1.28 (0.89)	22	2.37	ABCD
Social function	0.55 (0.43)	40	0.24 (0.53)	41	0.39 (0.41)	50	2.17 (0.59)	22	15.29	AC BC D
Medical treatment effects	0.57 (0.20)	41	0.11 (0.22)	42	0.51 (0.18)	51	0.16 (0.15)	24	10.87	AC BD
Instrument total	11.65 (3.54)	35	5.36 (3.27)	37	8.70 (2.18)	46	8.29 (2.94)	20	8.95	A B CD

The EQ-5D threshold (anchor) was set to 0.04. ANOVA was used to test for differences between age subgroups

.

## Appendix 3

See Table 8

**Table 8:** MID values estimated using the linear regression anchor-based method for each baseline health subgroup

GQLI domain	< 96 A		91–112 B		113–125 C		125–144 D		Largest *T*-statistic	Difference in means
	MID (SE)	*n*	MID (SE)	*n*	MID (SE)	*n*	MID (SE)	*n*		
Symptoms	9.51 (2.12)	36	4.83 (1.53)	39	2.75 (0.88)	36	− 1.53 (1.17)	33	30.43	A B C D
Emotions	3.41 (0.77)	36	1.93 (0.60)	40	1.73 (0.41)	36	0.44 (0.36)	34	22.07	A BC D
Physical function	3.64 (0.98)	36	1.18 (0.76)	40	1.50 (0.45)	34	− 0.73 (0.48)	34	25.76	A BC D
Social function	2.44 (0.63)	34	0.68 (0.50)	40	0.23 (0.42)	35	− 0.57 (0.32)	35	26.01	A B C D
Medical treatment effects	0.83 (0.21)	34	0.53 (0.23)	40	0.25 (0.16)	35	− 0.17 (0.14)	35	21.85	A B C D
Instrument total	20.00 (4.06)	34	9.08 (2.64)	39	6.46 (1.55)	34	− 2.06 (2.00)	31	32.39	A B C D

The EQ-5D threshold (anchor) was set to 0.04. ANOVA was used to test for differences between age subgroups

.

## Appendix 4

See Table 9

**Table 9:** Baseline GQLI domain scores and MID estimates for the overall cohort calculated using GQLI scores transformed to a scale of 0–100

GQLI domain	Baseline score		MID	
	Present study	Shi et al.	Present study	Shi et al.
Symptoms	77.41	76.59	6.72	6.42
Emotions	71.62	66.25	13.00	6.86
Physical function	66.63	64.61	7.57	7.64
Social function	80.66	55.25	6.57	6.46

MID estimates shown were estimated using the regression method

.

## References

[1] Revicki D, Hays RD, Cella D, Sloan J (2008). Recommended methods for determining responsiveness and minimally important differences for patient-reported outcomes. J Clin Epidemiol.

[2] Weldring T, Smith SMS (2013). Patient-reported outcomes (PROs) and patient-reported outcome measures (PROMs). Heal Serv Insights.

[3] Kingsley C, Patel S (2017). Patient-reported outcome measures and patient-reported experience measures. BJA Educ.

[4] Eypasch E, Williams JI, Wood-Dauphinee S, Ure BM, Schmülling C, Neugebauer E (1995). Gastrointestinal Quality of Life Index: development, validation and application of a new instrument. Br J Surg.

[5] Gurusamy KS, Davidson BR, McDonald JWD, Feagan BG, Jalan R, Kahrilas PJ (2019). Gallstone disease. Evidence-based gastroenterology and hepatology.

[6] Brazzelli M, Cruickshank M, Kilonzo M, Ahmed I, Stewart F, Mcnamee P (2014). Clinical effectiveness and cost-effectiveness of cholecystectomy compared with observation/conservative management for preventing recurrent symptoms and complications in adults presenting with uncomplicated symptomatic gallstones or cholecystitis: a syste. Health Technol Assess (Rockv).

[7] Mcdonald JWD, Feagan BG, Jalan R, Kahrilas PJ (2019). Evidence-based gastroenterology and hepatology.

[8] Mentes BB, Akin M, Irkorucu O, Tatlicioglu E, Ferahkose Z, Yildirim A (2001). Gastrointestinal quality of life in patients with symptomatic or asymptomatic cholelithiasis before and after laparoscopic cholecystectomy. Surg Endosc.

[9] Sandblom G, Videhult P, Karlson B, Wollert S, Darkahi B, Ljungdahl M (2007). Validation of gastrointestinal Quality of Life Index for assessment of gallstone-related symptoms. Value Health.

[10] Sandblom G, Videhult P, Karlson BM, Wollert S, Ljungdahl M, Darkahi B (2009). Validation of gastrointestinal quality of life index in Swedish for assessing the impact of gallstones on health-related quality of life. Value Health.

[11] Lien H-H, Huang C-C, Wang P-C, Chen Y-H, Huang C-S, Lin T-L (2007). Validation assessment of the Chinese (Taiwan) version of the Gastrointestinal Quality of Life Index for patients with symptomatic gallstone disease. J Laparoendosc Adv Surg Technol A.

[12] Hsueh L-N, Shi H-Y, Wang T-F, Chang C-Y, Lee K-T (2011). Health-related quality of life in patients undergoing cholecystectomy. Kaohsiung J Med Sci.

[13] Quintana JM, Arostegui I, Oribe V, de Tejada L, Barrios B, Garay I (2005). Influence of age and gender on quality-of-life outcomes after cholecystectomy. Qual Life Res.

[14] Fillingim RB, King CD, Ribeiro-Dasilva MC, Rahim-Williams B, Riley JLI (2009). Sex, gender, and pain: a review of recent clinical and experimental findings. J Pain.

[15] Wanjura V, Lundström P, Österberg J, Rasmussen I, Karlson BM, Sandblom G (2014). Gastrointestinal quality-of-life after cholecystectomy: indication predicts gastrointestinal symptoms and abdominal pain. World J Surg.

[16] Jaeschke R, Singer J, Guyatt GH (1989). Ascertaining the minimal clinically important difference. Control Clin Trials.

[17] Guyatt GH, Osoba D, Wu AW, Wyrwich KW, Norman GR, Aaronson N (2002). Methods to explain the clinical significance of health status measures. Mayo Clin Proc.

[18] King MT (2011). A point of minimal important difference (MID): a critique of terminology and methods. Expert Rev Pharmacoecon Outcomes Res.

[19] Willke R, Burke L, Erickson P (2004). Measuring treatment impact: a review of patient-reported outcomes and other efficacy endpoints in approved product labels. Control Clin Trials.

[20] Revicki DA, Cella D, Hays RD, Sloan JA, Lenderking WR, Aaronson NK (2006). Responsiveness and minimal important differences for patient reported outcomes. Health Qual Life Outcomes.

[21] Devlin N, Parkin D, Janssen B (2020). Methods for analysing and reporting EQ-5D data.

[22] Shi H-Y, Lee K-T, Lee H-H, Uen Y-H, Na H-L, Chao F-T (2009). The minimal clinically important difference in the Gastrointestinal Quality-of-Life Index after cholecystectomy. Surg Endosc.

[23] Sutherland J, Liu G, Crump T, Bair M, Karimuddin A (2018). Relationship between preoperative patient-reported outcomes and hospital length of stay: a prospective cohort study of general surgery patients in Vancouver, Canada. J Health Serv Res Policy.

[24] Sutherland JM, Crump RT, Chan A, Liu G, Yue E, Bair M (2016). Health of patients on the waiting list: opportunity to improve health in Canada?. Health Policy (New York).

[25] Lien HH, Huang CC, Wang PC, Huang CS, Chen YH, Lin TL (2009). Changes in quality-of-life following laparoscopic cholecystectomy in adult patients with cholelithiasis. J Gastrointest Surg.

[26] EuroQoL Group (1990). EuroQol-a new facility for the measurement of health-related quality of life. Health Policy (New York).

[27] Brooks R (1996). EuroQol: the current state of play. Health Policy.

[28] Dolan P (1997). Modeling valuations for EuroQol health states. Med Care.

[29] Dolan P (1996). The effect of experience of illness on health state valuations. J Clin Epidemiol.

[30] Bansback N, Tsuchiya A, Brazier J, Anis A (2012). Canadian valuation of EQ-5D health states: preliminary value set and considerations for future valuation studies. PLoS ONE.

[31] Charlson ME, Pompei P, Ales KL, MacKenzie CR (1987). A new method of classifying prognostic comorbidity in longitudinal studies: development and validation. J Chronic Dis.

[32] Lydick E, Epstein RS (1993). Interpretation of quality of life changes. Qual Life Res.

[33] Norman GR, Sloan JA, Wyrwich KW (2003). Interpretation of changes in health-related quality of life the remarkable universality of half a standard deviation. Med Care.

[34] Coretti S, Ruggeri M, McNamee P (2014). The minimum clinically important difference for EQ-5D index: a critical review. Expert Rev Pharmacoecon Outcomes Res.

[35] Copay AG, Subach BR, Glassman SD, Polly DW, Schuler TC (2007). Understanding the minimum clinically important difference: a review of concepts and methods. Spine J.

[36] Sloan JA, Cella D, Hays RD (2005). Clinical significance of patient-reported questionnaire data: another step toward consensus. J Clin Epidemiol.

[37] Rejas J, Pardo A, Ruiz MÁ (2008). Standard error of measurement as a valid alternative to minimally important difference for evaluating the magnitude of changes in patient-reported outcomes measures. J Clin Epidemiol.

[38] Schünemann HJ, Griffith L, Jaeschke R, Goldstein R, Stubbing D, Guyatt GH (2003). Evaluation of the minimal important difference for the feeling thermometer and the St. George’s Respiratory Questionnaire in patients with chronic airflow obstruction. J Clin Epidemiol.

[39] Puhan MA, Frey M, Büchi S, Schünemann HJ (2008). The minimal important difference of the hospital anxiety and depression scale in patients with chronic obstructive pulmonary disease. Health Qual Life Outcomes.

[40] Simon AS, Neary MP, Cella D (2007). Estimation of minimally important differences in EQ-5D utility and VAS scores in cancer. Health Qual Life Outcomes.

[41] McClure N, Sayah F, Xie F, Luo N, Johnson J (2017). Instrument-defined estimates of the minimally important difference for EQ-5D-5L Index Scores. Value Health.

[42] Le QA, Doctor JN, Zoellner LA, Feeny NC (2013). Minimal clinically important differences for the EQ-5D and QWB-SA in Post-traumatic Stress Disorder (PTSD): results from a doubly randomized preference trial (DRPT). Health Qual Life Outcomes.

[43] Walters SJ, Brazier JE (2005). Comparison of the minimally important difference for two health state utility measures: EQ-5D and SF-6D. Qual Life Res.

[44] Team RC (2017). R: a language and environment for statistical computing.

